# Molecular mapping of candidate genes in determining red color of perilla leaf

**DOI:** 10.1007/s44307-025-00058-8

**Published:** 2025-02-14

**Authors:** Guanwen Xie, Yuxuan Zhang, Shen Xiao, Duan Wu, Hongbin Wang, Qi Shen

**Affiliations:** Institute of Medical Plant Physiology and Ecology, School of Pharmaceutical Sciences, Guangzhou University of Chinese Medicine, Guangzhou, 510006 China

**Keywords:** *Perilla frutescens*, Anthocyanin, BSA-seq, BSR-seq, Regulation mechanism, Transcription factors

## Abstract

*Perilla frutescens* is a traditional medicinal plant and functional food in Asian communities, characterized by distinct red and green leaf types that have significant phenotypic and medicinal implications. However, the genetic mechanisms controlling anthocyanin synthesis in this species remain unclear. Genetic analysis serves as a powerful tool for investigating the pivotal genes and regulatory mechanisms governing anthocyanin accumulation in red and green perilla. In this study, an F2 segregation population was constructed from a hybrid of red and green perilla, and representative samples were subjected to mix-sequencing using BSA-seq and BSR-seq. A 6.0 Mb candidate region on chromosome 8 was identified, pinpointing *PfMYB113b*, *PfC4H1*, and PfF3H as key genes involved in anthocyanin biosynthesis. The insertion of a repeat sequence in the promoter of *PfMYB113b* leads to alterations in gene expression levels. Furthermore, *PfMYB113b* regulates the transcription of *PfC4H1* and *PfF3H*, thereby influencing anthocyanin synthesis. These findings enhance our understanding of the genetic regulatory mechanisms underlying leaf coloration in perilla.

**Supplementary Information:**

The online version contains supplementary material available at 10.1007/s44307-025-00058-8.

## Introduction

*Perilla frutescens* (L.) Britt., a member of the *Lamiaceae* family (Pandey & Bhatt 2008; Zhou, XJ et al*.*, 2014), is extensively cultivated in China, Japan, and Korea (Lee & Ohnishi 2003). This plant serves a significant role as both a medicinal and culinary resource in China, with various medical and nutritional applications (Liu et al. 2013). Its leaves are versatile, functioning as a medicinal herb, vegetable, and spice (Meng et al. 2008; Ha et al. 2012a, b, c; Ahmed, M et al*.*, 2018). Extracts from perilla leaves contain phenolic acids, volatile oils, flavonoids, and amino acids, exhibiting pharmacological effects such as antioxidative, antibacterial, and anti-inflammatory properties, as well as cough relief (Igarashi & Miyazaki 2013; Ahmed, HM, 2018). Furthermore, Perilla has been traditionally used in herbal medicine to alleviate symptoms associated with cough and certain intestinal disorders (Yang et al. 2012; Ha et al. 2012a; Ha et al. 2012b; Zhou, X et al*.*, 2014; Yu et al. 2017).

Perilla can be classified into red-leaf and green-leaf chemotypes based on leaf color. The anthocyanin present in the red chemotype is widely used as a food coloring agent, while red perilla is often valued as an ornamental plant in gardens due to its extensive morphological diversity and appealing aesthetics (Pandey & Bhatt 2008). The red hue of perilla is primarily attributed to the presence of malonylshisonin (3-O-(6-O-(E)-p-coumaryl-β-D-glucopyranosyl)-5-O-(6-O-malonyl-β-D-glucopyranosyl)-cyanidin) (Honda et al. 1994; Saito & Yamazaki 2002; Meng et al. 2006). Anthocyanins have garnered significant attention in recent years for their potential in preventing neurological and cardiovascular disorders, cancer, diabetes, and age-related degenerative diseases in humans (Butelli et al. 2008; Sun et al. 2011, 2012, 2013). Furthermore, perilla anthocyanins exhibit potent antioxidant activity, including reducing ability, DPPH free radical scavenging, superoxide anion scavenging, hydrogen peroxide scavenging, and metal chelating properties (Gülçin et al. 2005).

The biosynthesis and regulation of anthocyanins have been extensively studied (Saito & Yamazaki 2002; Stracke et al. 2007). The MYB-bHLH-WD40 (MBW) complex serves as a crucial transcriptional regulator in flavonoid biosynthesis (Xu et al. 2015). MYB proteins are classified into four groups based on the number and position of MYB domain repeats: 1R-MYB/MYB-related, R2R3-MYB, 3R-MYB, and 4R-MYB (Yan et al. 2021). Notable examples of R2R3-MYB transcriptional activators include MYB75/anthocyanin pigment 1 (PAP1), MYB90/PAP2, MYB113, and MYB114 in *Arabidopsis thaliana* (Gonzalez et al*.*, 2008a), MdMYB10 in apple (Espley et al. 2009), SlAN2-LIKE in tomato (Sun et al. 2020), PyMYB10 in pear (Qian et al. 2014), DcMYB7 in carrot (Xu et al. 2019), and Ruby1 in orange (Butelli et al. 2012). The MYB113 transcription factor, classified under SG6, is recognized for its role in regulating anthocyanin biosynthesis (Dubos et al. 2010).

Bulk Segregant Analysis (BSA) was initially introduced as a method for identifying molecular markers associated with downy mildew resistance loci in lettuce (Michelmore et al. 1991). The mixed pool method significantly reduces the workload and costs associated with genotyping individual plants while enhancing the screening efficiency of linked markers, making it essential for gene mapping in segregating populations (Michelmore et al. 1991; Li & Xu, 2022a). Furthermore, BSA mapping has evolved from focusing on qualitative traits to encompassing quantitative traits controlled by major genes (Salunkhe et al. 2011; Aoun et al. 2017; Zongo et al. 2017; Li & Xu, 2022b). Recently, the integration of BSA, next-generation sequencing (NGS), and bioinformatics analysis has led to the development of more effective methods, including QTL-seq, MutMup, MutMup + , and BSR-seq. Bulked Segregant RNA-Seq (BSR) is an analytical method that combines BSA with RNA-Seq (Liu et al. 2012). These methods have been successfully applied to localize Quantitative Trait Loci (QTL) and identify candidate genes in various crops (Abe et al. 2012; Fekih et al. 2013; Takagi et al. 2013; Steuernagel et al. 2016).

In this study, we established a segregating population by crossing red-leaf and green-leaf chemotypes. The pools containing leaves with intense purple and green colors were sequenced separately. Both BSA and BSR were conducted to identify the genomic regions and key genes. The putative regulatory mechanisms were subsequently verified. This genetic regulatory investigation will enhance our understanding of anthocyanin biosynthesis and regulation in perilla.

## Results

### Phenotypic characterization and genetic analysis of the perilla population

To investigate the genetic characteristics of leaf color phenotypes in perilla, we selected the red perilla cultivar “PA21” as the female parent and the green perilla cultivar “M84” as the male parent to construct a genetic population. The F1 generation plants exhibited the red phenotype, which were then self-crossed to produce the F2 generation. The leaf colors of the F2 population displayed clear segregation.

The leaf colors of the F2 population lines were subjected to further statistical analysis at two experimental sites (Table 1). In the Guiyang (2019) experimental site, among the 554 F2 lines, 127 lines displayed green leaf color, 309 lines exhibited an intermediate red color, and 118 lines showed self-colored leaves. This distribution aligned with the expected segregation ratio of 1:2:1, with a Chi-square test yielding a value of 0.19. Similar results were observed in the Guangzhou (2021) experimental site. Among the 106 F2 plants in the test populations, 25 had green leaf color on both sides, 45 exhibited an intermediate purple and green color, and 36 displayed self-colored leaves on both sides. The Chi-square test yielded a value of 0.21. These findings suggest that the leaf color of perilla is regulated by dominant and recessive major genes.

**Table 1:** Genetic analysis of purple frontal leaf in genetic population

Time	Population	Scale	Green	Medium	Purple	Expected ratio	χ2
2019 (Pop1, Guiyang)	M84 x PA21	554	127	309	118	1:02:01	0.19
2021 (Pop2, Guangzhou)	PA21 x M84	106	25	45	36	1:02:01	0.21

Expected ratio: Dominant homozygous: Heterozygous: Recessive homozygous = 1:2:1

Expected ratio: Dominant trait individual: Recessive trait individual = 3:1

χ2: Chi-square test value. χ2 < 3.84 is considered as significant correlated

*p* value: *p* value > 0.05 indicate no statistically significant differences

**Fig. 1:**
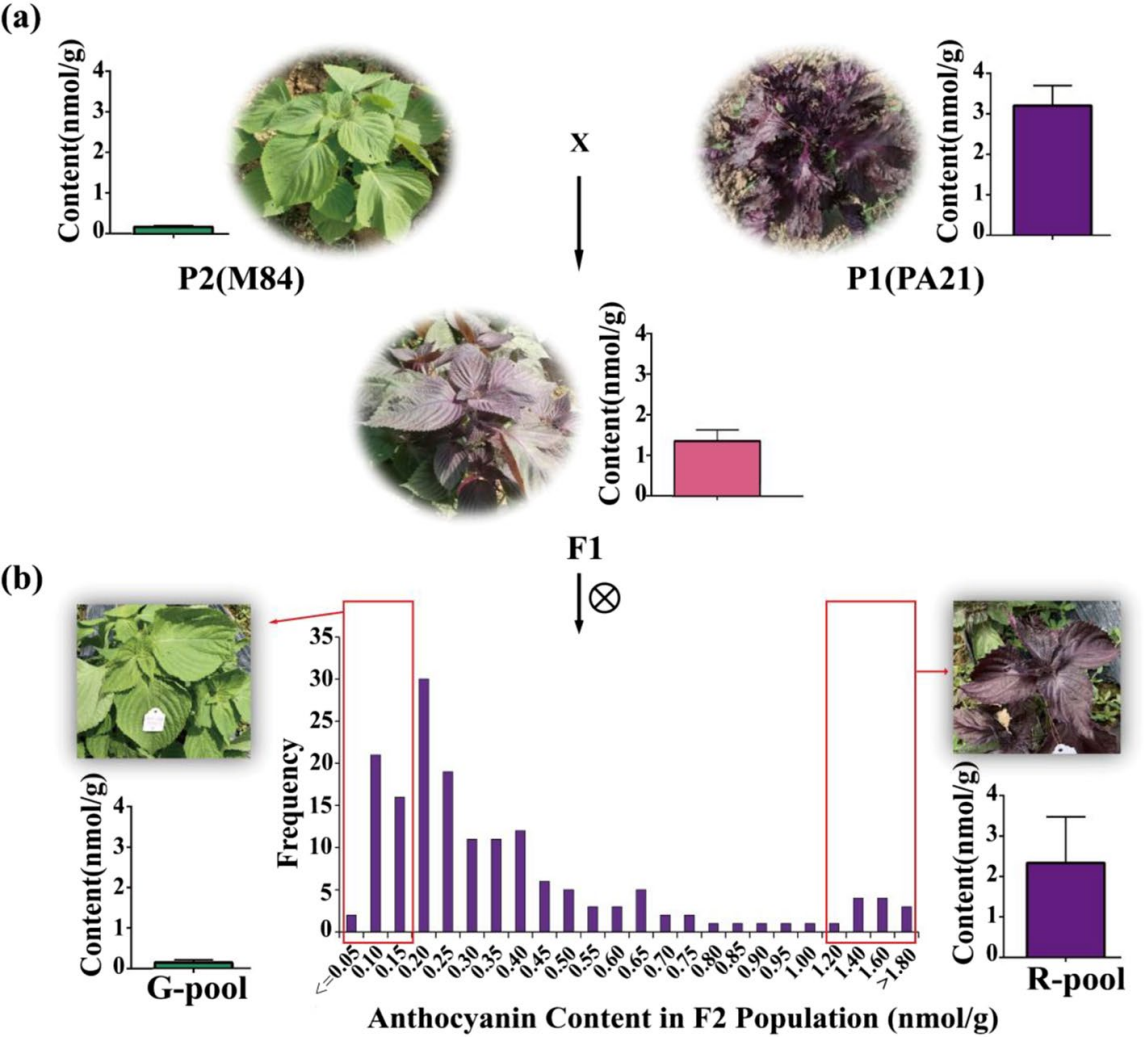
Phenotype and anthocyanin content of parents and F2 lines in perilla population. **a** Phenotypes and anthocyanin contents of low-value parents M84 (P2), F1 progeny and high value parents PA21 (P1). **b** The frequency of the anthocyanin contents in the F2 generation populations. Phenotype and anthocyanin content of low-value mixed pool generation (G-pool) and high-value mixed pool generation (R-pool)

The total anthocyanin content in the parental lines and F2 population leaves was further analyzed. PA21 exhibited significantly higher total anthocyanin content than M84. The F1 hybrid lines displayed intermediate total anthocyanin content between the two parents (Fig. 1a). In the F2 generation, the anthocyanin contents showed a skewed distribution, indicating notable disparities among plants with varying anthocyanin levels (Fig. 1b). Leaves with both high and low anthocyanin contents were selected to create mixing pools for BSA and BSR analyses, respectively (Fig. S1).

### BSA-seq analysis of perilla population

Whole-genome resequencing was performed on PA21 as the high-value parent, M84 as the low parent, while 30 green F2 lines were equally mixed to form the R-pool and 30 red F2 lines constituted the G-pool. A total of 111.36 GB of raw data was generated with high sequencing quality (Q20 ≥ 96.48%, Q30 ≥ 90.64%), resulting in 110.91 GB of clean data after filtering. The GC content ranged from 35.69% to 36.05% (Table S1, Fig. S2). The alignment rate of clean reads mapping to the perilla genome ranged from 98.77% to 99.52% (Table S2).

**Fig. 2:**
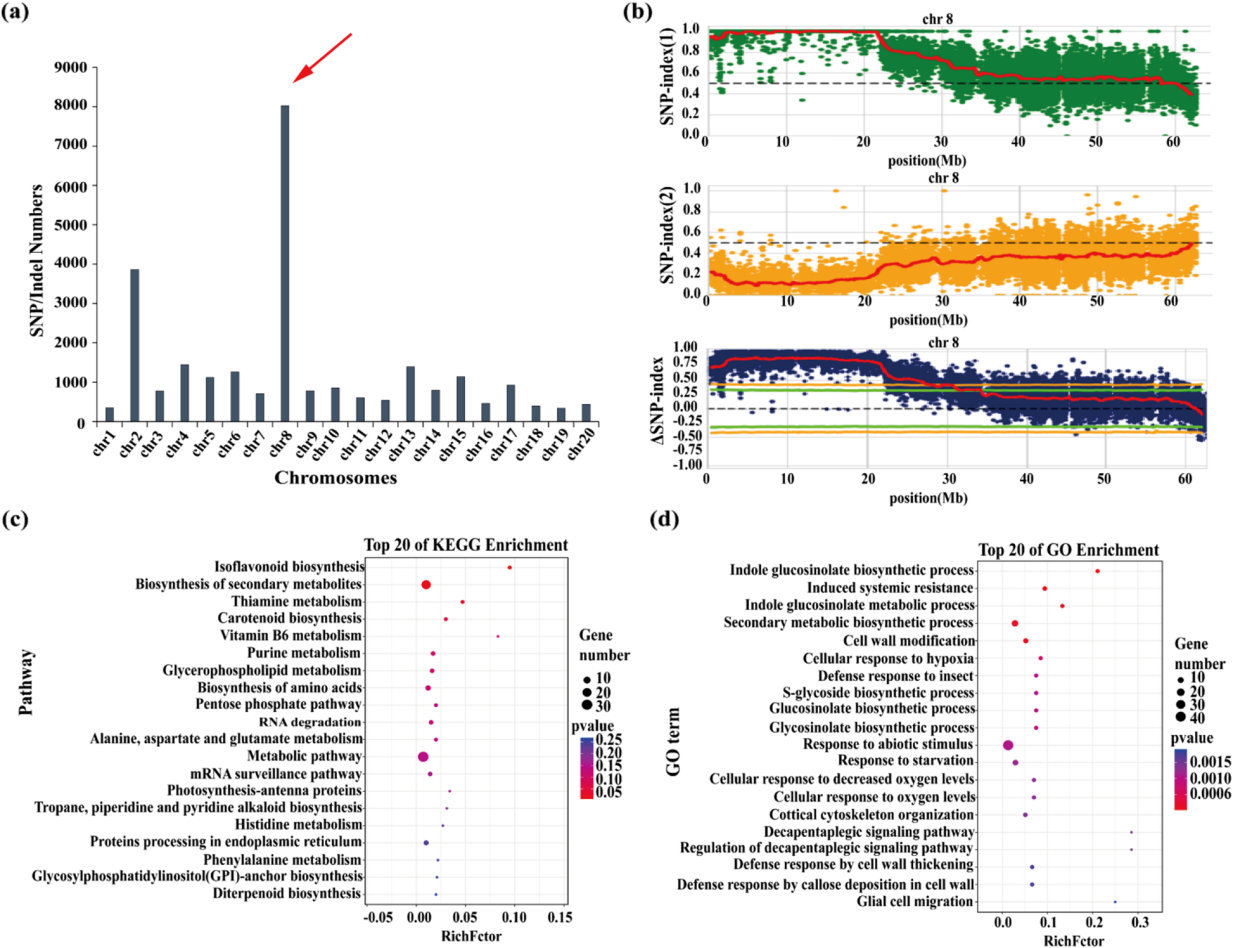
SNP identification and variation region determination in BSA-Seq. **a** Significant variation of SNP/Indel sites in 20 chromosomes. **b** The significant variation in chromosome Chr8. **c** KEGG pathway enrichment of the DEGs in variation region. **d** The top-20 GO enrichment of the DEGs in variation region

A total of 6,846,276 SNP polymorphic sites and 1,414,655 Indel polymorphic sites were identified (Table S3). After data filtering, the high-value parent PA21 was selected as the reference parent, and the 238,808 SNP marker sites between the two pools were analyzed. The variations were unevenly distributed across chromosomes, with Chr8 exhibiting the highest number of SNP sites (Fig. 2a). Using the Δ(SNP/Indel-index) method with a 95% confidence interval, the candidate variation region was determined to range from 1.80 to 21.80 Mb on Chr8 (Fig. 2b, Fig. S3).

The analysis of variant types on Chr8 revealed a significant presence of intronic, missense, and synonymous variants. A total of 101 genes exhibited missense variants within the variation region (Table S4). The Kyoto Encyclopedia of Genes and Genomes (KEGG) analysis was enriched in isoflavonoid biosynthesis, secondary metabolite biosynthesis, and phenylalanine metabolism pathways. Similarly, the Gene Ontology (GO) analysis was enriched in the secondary metabolic biosynthesis process (Fig. 2c, d).

### BSR-Seq of perilla population

**Fig. 3:**
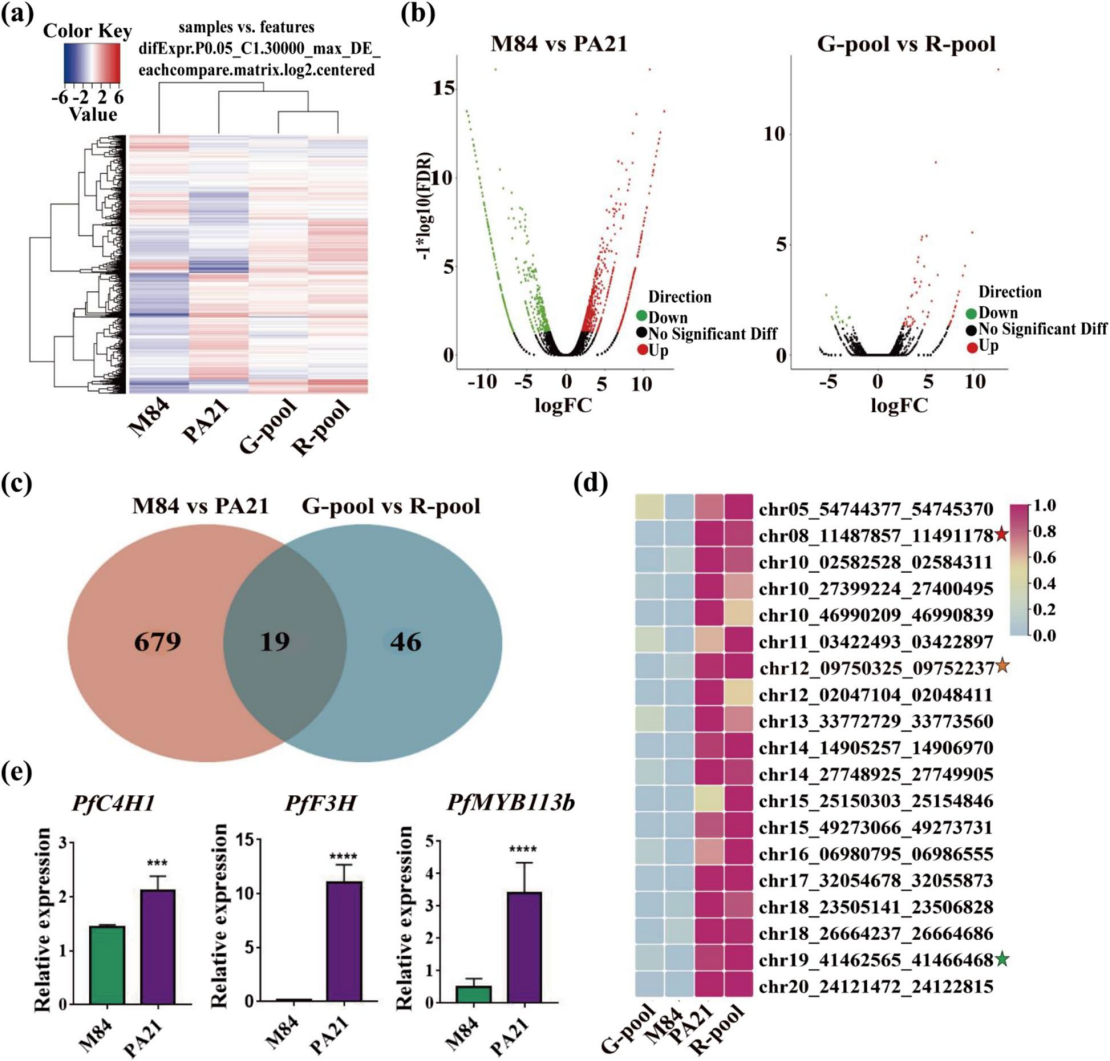
Mining and functional verification of differently expressed genes in BSR-Seq. **a** Differently gene expression the transcriptome. The middle grid is normalized to the level of gene expression between 0 and 1. **b** Scatter diagram of the DEG. The green dots represent downregulated genes, the red dots represent upregulated genes and the black dots represent non-differentially expressed genes. **c** Wenn diagram of cross-concentrated up-regulated DEG in PA21 and R-pool compared M84 and G-pool. **d** Heat map of DEG between BSA-Seq and BSR-Seq data. The red star marker is *PfMYB113b*, the yellow star marker is *Pfc4HI*, and the green star marker is *PfF3H*. **e** qRT-PCR analysis of candidate genes *PfMYB113b*, *Pfc4HI* and *PfF3H*

To validate gene expression, the BSR-Seq was employed based on transcriptome sequencing (Fig. S4a). A total of 66,197,001 clean reads, comprising 9,870,164,378 clean bases were generated (Table S5). The mapping rate of the reads to the reference genome reached 99% (Table S6). Principal component analysis (PCA) unveiled notable distinctions among samples from various groups (Fig. S5a, b). The expression levels exhibited a strong correlation within each duplicate sample (Fig. S5c-e). Comparative analysis indicated that M84 had a higher number of differentially expressed genes (DEGs) compared to PA21, while the number of DEGs between the G-pool and R-pool was lower (Fig. 3a, b). Enrichment analyses of DEGs revealed that molecular functions associated with nucleic acid binding, transcription factor activity, and cellular components were significantly enriched (Fig. S6a, b). There was predominant enrichment in the KEGG pathways linked to phenylalanine biosynthesis, flavonoid biosynthesis, glutathione metabolic processes, and anthocyanin-containing compound biosynthesis among these DEGs (Fig. S7). These observations suggest that the biosynthesis of anthocyanins differs significantly between M84 and PA21, as well as between the G-pool and R-pool. Notably, Chr8 was identified as a significant region linked to the color variation of perilla leaves in the mixed-pool sequencing data, specifically spanning from 11.00 to 17.30 Mb (Fig. S4b).

### The identification of candidate genes combining the BSA-Seq and BSR-Seq

By integrating the BSA-Seq and BSR-Seq data, 19 genes exhibited consistent differential expression between M84 and PA21, as well as between the G-pool and R-pool (Fig. 3c, d). It is well established that the MYB113 transcription factor plays a crucial role in the positive regulation of flavonoid and anthocyanin bioaccumulation, while Cinnamate-4-hydroxylase (C4H) and Flavonoid-3-hydroxylase (F3H) serve as key catalytic enzymes in anthocyanin biosynthesis. Noteworthy, *PfMYB113b* (chr08_11487857_11491178) exhibited significantly higher expression levels in PA21 and the R-pool (Fig. 3d, Table S7). Our previous work indicated that Myb113 genes are important regulators of anthocyanin accumulation in *Perilla* (Zhang et al. 2021), and PfMYB113b shares 60% homology with Myb113 genes (Fig. S8). Additionally, *PfC4H1* (chr12_09750325_09752237) and *PfF3H* (chr19_41462565_41466468), both involved in the anthocyanin biosynthesis pathway, also exhibited elevated expression levels in PA21 and the R-pool, respectively (Fig. 3d, Table S7, S9). The expression levels of PfMYB113b, PfC4H1, and PfF3H were further confirmed to be significantly higher in PA21 compared to M84 using qRT-PCR (Fig. 3e).

### *PfMYB113b* regulates the expression of *PfF3H* and *PfC4H1* genes to promote anthocyanin accumulation

**Fig. 4:**
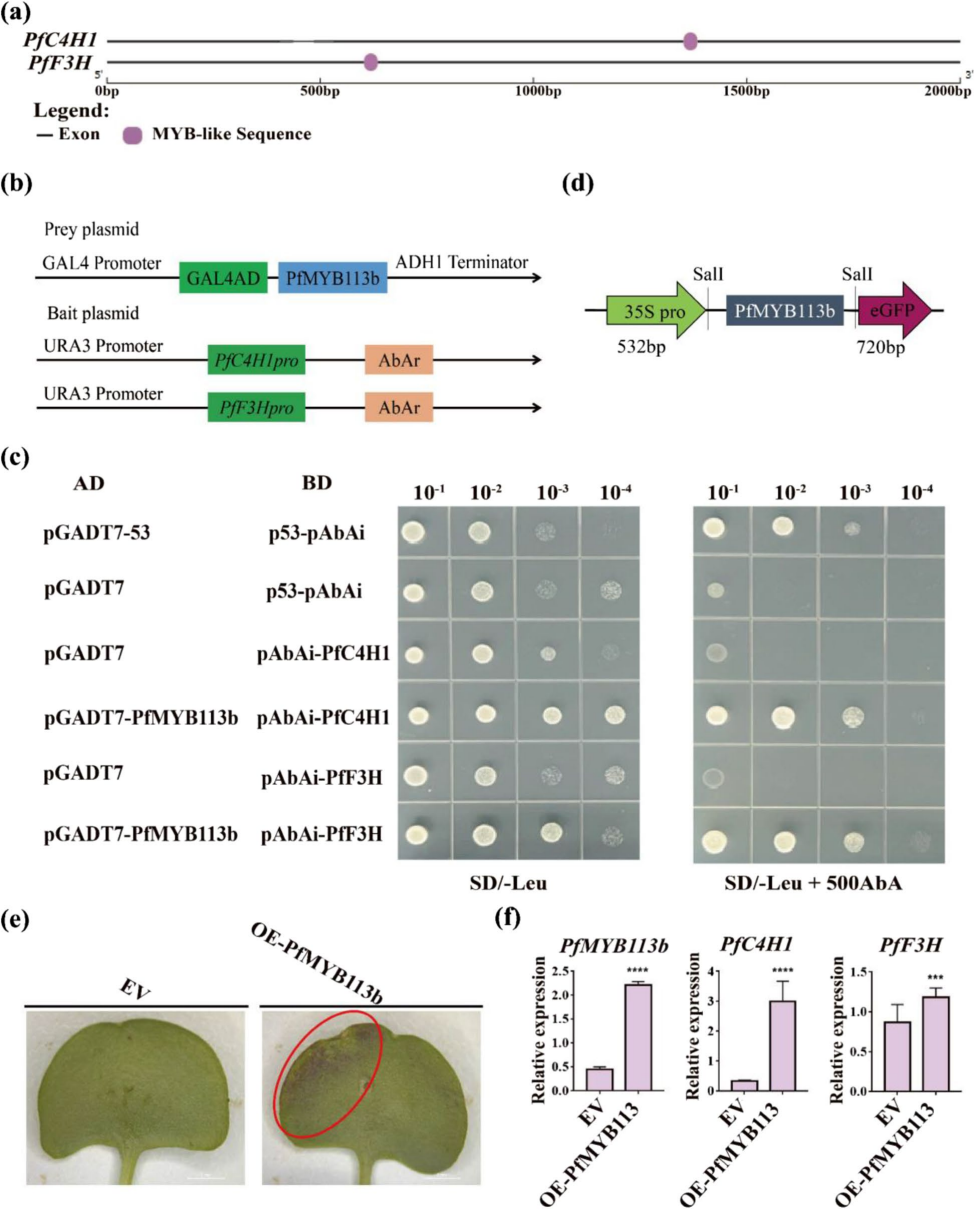
Functional analysis of *PfMYB113b*. **a** Cis-acting element analysis of *PfC4HI* and *PfF3H* promoters. **b** Schematic diagram of Y1H experimental vector. **c** *PfMYB113b* could activate promoters of PfC4HI and PfF3H in Y1H experiment. **d** Schematic diagram of overexpressed vector. **e** *PfMYB113b* immediately transferred resulted in cotyledon turn red in Perilla. **f** The expression analysis of *PfMYB113b*, *PfC4HI* and *PfF3H* genes *PfMYB113b* immediately transferred tissues

To investigate the regulatory relationship between *PfMYB113b*, *PfC4H1*, and *PfF3H*, we analyzed the cis-acting elements within the promoters of *PfC4H1* and *PfF3H*. The promoters contained binding elements such as the MYB-like sequence (TAACCA), indicating a potential regulatory role of MYB transcription factors on the *PfC4H1* and *PfF3H* genes (Fig. 4a). Subsequently, the coding sequence (CDS) of *PfMYB113b* was successfully cloned and inserted into the prey plasmid pGADT7 (Fig. S8 and Fig. 4b). Moreover, fragments of *PfC4H1*pro (-1000 ~ -500 bp) and *PfF3H*pro (-1700 ~ -1200 bp) were synthesized and integrated into the bait plasmid pAbAi (Fig. 4b). Thereafter, the yeast one-hybrid system (Y1H) was utilized to validate the interaction between PfMYB113b and both *PfC4H1* and *PfF3H*. The results demonstrated growth of the pAbAi-*F3H* + pGADT7-MYB113b-JG and pAbAi-*C4H* + pGADT7-MYB113b-JG combinations on the SD/-Leu plate, indicating successful transformation of the AD plasmid. In the presence of 500 ng/ml AbA, pGADT7-MYB113b-JG exhibited growth with the pAbAi-C4H and pAbAi-F3H combinations, similar to the positive control combination of p53-pAbAi + pGADT7-53. These outcomes suggest that PfMYB113b can activate *PfF3Hpro* and *PfC4H1pro* (Fig. 4c).

To explore the role of *PfMYB113b* in anthocyanin synthesis in perilla, we cloned *PfMYB113b* into the pCAMBIA2300-eGFP vector and conducted transient transformation using the Agrobacterium system on the cotyledons of perilla seedlings (Fig. 4d). The empty vector served as the negative control. The findings revealed that after 7 days of treatment, the green perilla cotyledons accumulated anthocyanins, exhibiting a purplish-red hue (Fig. 4e). Moreover, the expression level of *PfMYB113b* showed significant upregulation (Fig. 4f), indicating its potential to enhance anthocyanin synthesis in perilla leaves. Additionally, the expression levels of *PfC4H1* and *PfF3H* were notably elevated in the cotyledons with anthocyanin accumulation (Fig. 4f), suggesting that PfMYB113b may promote anthocyanin accumulation in perilla by modulating the transcription of *PfC4H1* and *PfF3H*.

### Variants in *PfMYB113b* promoter effect gene expression and anthocyanin accumulation

**Fig. 5:**
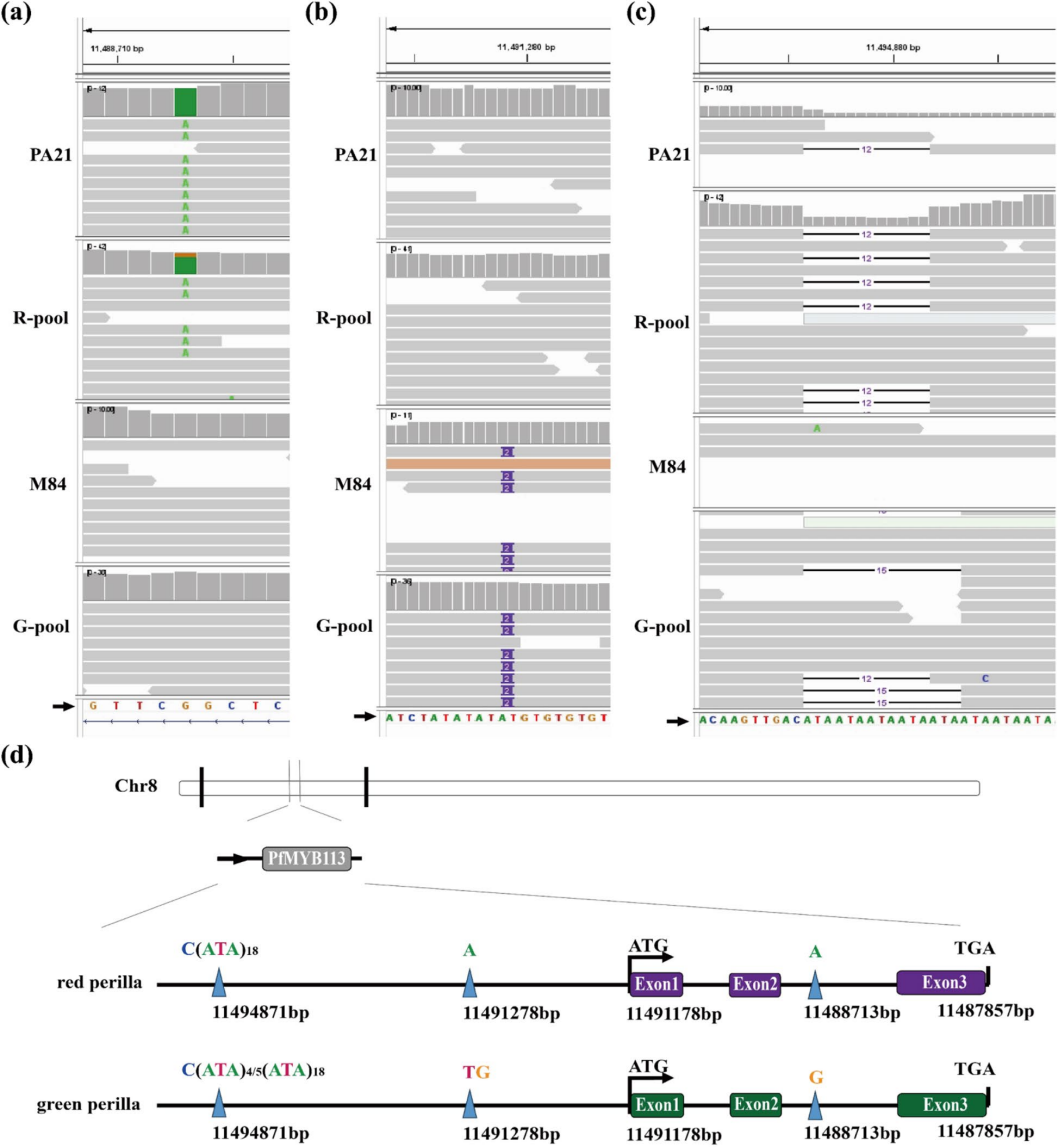
Variation site analysis of *PfMYB113b* promoter. **a** and (**b**) Base information differentitation at P1/ R-pool and P2/G-pool. **c** The 12 short repeat insertions in upstream region. **d** Illustration of the variation of *PfMYB113b* gene red and green perilla

The insertion of long terminal repeats (LTRs) upstream of Myb113 has been reported to influence gene expression and color formation (Zhang et al. 2021). In our research, we investigated the upstream region of *PfMYB113b* (chr08_11487857_11491178), a homologous protein in the allotetraploid perilla genome. Interestingly, we identified variations in the promoter of *PfMYB113b* on chromosome 8, while no significant variation was found in the coding region. Specifically, two variations in the upstream region of *PfMYB113b* were identified: a 2-base insertion and the deletion of an (ATA)_4/5_ tandem repeat in the upstream 5 kb region of the *PfMYB113b* gene (Fig. 5b). Remarkably, this deletion site of the repeat was heterozygous in the red parent and homozygous in the green parent. Statistical analysis indicated a ratio of intact to missing reads of approximately 1:1 in the R-pool and 2:1 in the G-pool (Fig. 5c, d). These results suggest that the absence of this sequence potentially impacts *PfMYB113b* expression, further influencing the regulation of genes in the anthocyanin biosynthesis pathway and the development of red leaf color traits.

**Fig. 6:**
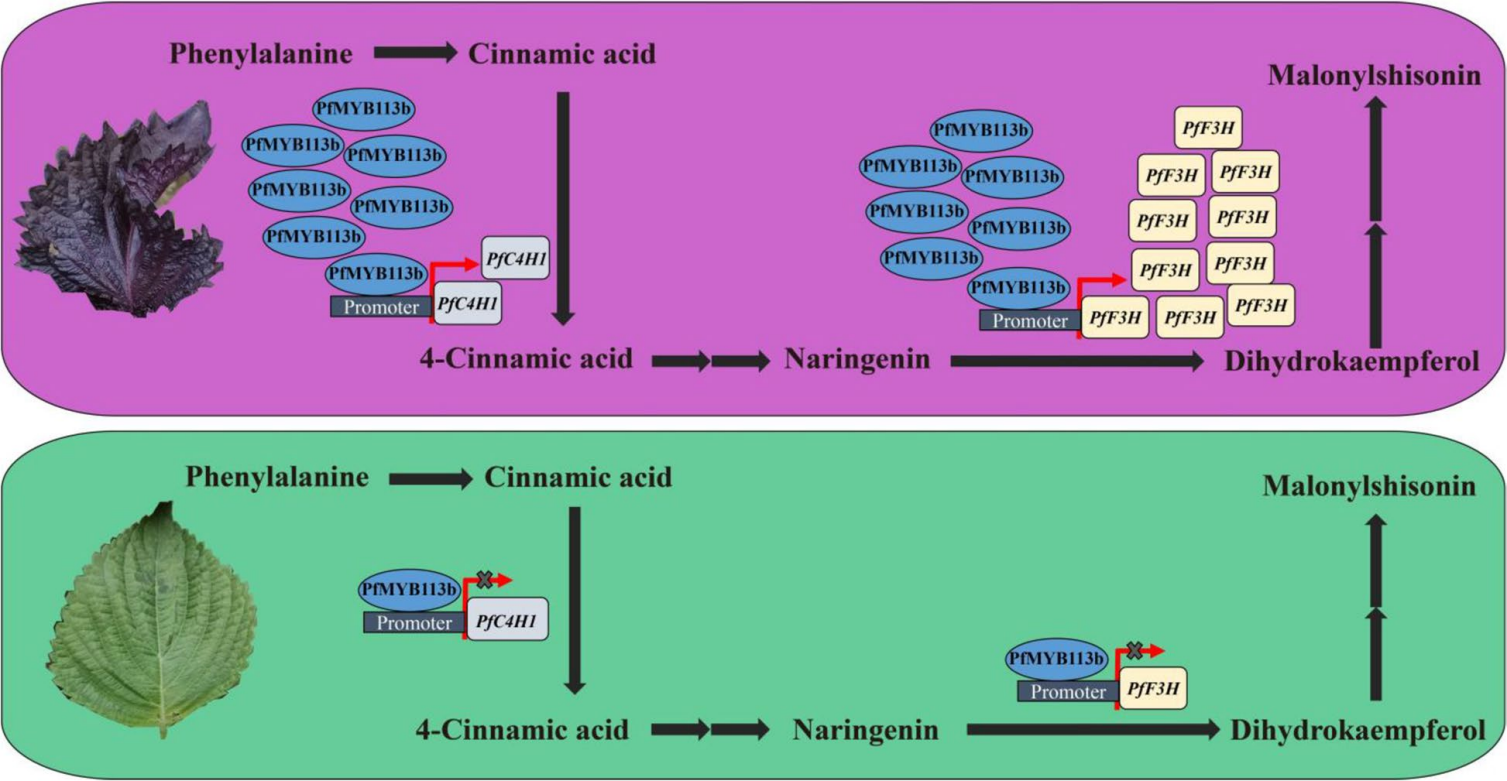
Model diagram of PfMYB113b regulating anthocyanin synthesis in perilla. The transcriptional level of *PfMYB113b* is significantly higher in red perilla, which activated the two key anthocyanin biosynthesis genes, *PfC4H1* and *PfF3H*, leading to the accumulation of anthocyanins in perilla

In conclusion, this study presents a regulatory mechanism model for anthocyanin biosynthesis in red and green perilla (Fig. 6). Based on the integrated analysis of BSA-Seq and BSR-Seq, *PfMYB113b*, *PfC4H1*, and *PfF3H* were identified as important genes involved in anthocyanin accumulation, located on chromosome 8, and showed significantly higher expression levels in the red parent and the R-pool. The transcriptional level of *PfMYB113b* is markedly higher in red perilla, which is potentially influenced by variations in the upstream promoter, activating the two key anthocyanin biosynthesis genes, *PfC4H1* and *PfF3H*, leading to the accumulation of anthocyanins in perilla.

## Discussion

*Perilla frutescens* is widely utilized as a traditional medicine and functional food in Asian communities (Igarashi & Miyazaki 2013). Previous genetic studies on pigment formation in perilla have revealed that three distinct loci promote anthocyanin production (Koezuka et al. 1985). Gene A governs pigmentation in both the leaf and stem, while the presence of the dominant gene K is necessary for pigmentation in the upper and lower epidermis of the leaf. Additionally, gene B exclusively promotes pigmentation in the stem (Yamazaki et al. 2003; Yamazaki & Saito 2011). Myb-P1 and Myb-C05 are among the MYB transcription factors that regulate chemotype-specific anthocyanin production (Gong et al. 1999; Springob et al. 2003). The expression of Myb-P1 was ten times higher in the leaves of the red form compared to the green form. The Myb-C05 gene is exclusively expressed in the red form and forms the MBW complex with MYC-RP and PFWD, similar to the TT2-GL3/EGL3-TTG1 complex in Arabidopsis (Dubos et al. 2010).

MYB113 belongs to the R2R3-MYB transcription factors and has been reported as a key regulator of anthocyanin biosynthesis in various plant parts (Dubos et al. 2010). The overexpression of AtMYB113 in Arabidopsis significantly enhances pigment production (Gonzalez et al*.*, 2008b; Li et al. 2014). In eggplant, SmMYB113 contributes to anthocyanin synthesis and accumulation under light induction (Li et al. 2017; Zhou et al. 2020). Ectopic expression of PcMYB113 in Arabidopsis thaliana induces anthocyanin accumulation in the seed coat, cotyledon, and mature leaves (Song et al. 2021). GbBM, which encodes the R2R3 MYB113 transcription factor, directly regulates the promoters of four flavonoid biosynthesis genes, controlling purple spot formation at the base of flower petals in the cultivated tetraploid cotton species Gossypium barbadense (Abid et al. 2022).

In previous studies, the GWAS method was used to investigate the pseudo-gene *Myb113* as an important transcription factor involved in leaf color in perilla. Complete copies of *Myb113* were found in both crispa and diploid materials with green leaves, but the diploid inactivated the red leaf phenotype through Gypsy-type retrotransposon insertion prior to polyploid development. A 9967-bp fragment deletion in the 3’ region of the LTR element upstream of *Myb113* was identified in all red lines, suggesting that the red leaf phenotype was restored by partial removal of the 3’ end LTR of *Myb113* in the tetraploid crispa branch (Zhang et al. 2021). In this study, we observed a significantly higher expression level of homozygous *PfMYB113b* in the red form compared to the green form, leading to increased anthocyanin accumulation after the immediate expression of *PfMYB113b* in the cotyledon of perilla. Additionally, *PfMYB113b* was found to regulate the transcription levels of two crucial genes involved in anthocyanin synthesis, namely *PfC4H1* and *PfF3H*. Based on these findings, we propose that *PfMYB113b* may be the gene responsible for perilla pigmentation. We also identified three coding mutations: an in-frame deletion of exon 2 (6 bp), a deletion of exon 3 (1 bp), and a non-significant SNP. Furthermore, this study discovered that another PfMYB113b plays a crucial regulatory role in the red leaf phenotype of perilla and identified a difference in the number of repeats of the short segment (ATA)n in the upstream region of the red-green *PfMYB113b* promoter. Hence, the reduction in the number of (ATA)n short segment repeats leads to the transcriptional activation of the *PfMYB113b* allele, thereby increasing its transcription level and inducing the expression of *PfC4H1* and *PfF3H*, ultimately promoting anthocyanin accumulation.

## Materials and method

### Plant materials and phenotype determination

The varieties “PA21” and “M84” were initially cultivated by the research group during early breeding efforts. These sister lines exhibit a high level of consistency in agronomic traits and genetic background, albeit with notable morphological differences. Over several years, a site phenotype survey revealed the presence of both positive and negative leaf colors in purple, as well as leaf folds. In contrast, M84 displayed both positive and negative leaf colors in green, along with flat leaves (Fig. 1). Subsequently, the F2 population was generated by crossing a single F1 individual, with PA21 as the female parent and M84 as the male parent. All entries were sown in the growth matrix during the years 2019 and 2020. The resulting seedlings were then transplanted outdoors in Guizhou Province and Guangzhou, where they were grown for a period of 60 days with row spacing set at 50 cm. Leaf samples were collected prior to flowering, ensuring simultaneous collection.

### Genomic DNA isolation and bulking

Based on the phenotypic assessment, we selected 30 individuals exhibiting red leaves and another 30 individuals with green leaves from the F2 population. These individuals were chosen to create extreme phenotype bulks for BSA-seq analysis. Genomic DNA was extracted from fresh leaves of both the F2 individuals and the parents using a DNA Secure Plant Kit (Tiangen, Beijing, China). The concentration and quality of the DNA were assessed using an ND-1000 spectrophotometer (NanoDrop, Wilmington, DE, USA) and through electrophoresis on a 1.0% agarose gel with standard lambda DNA. Two bulk samples, designated as the G-pool and the R-pool, were created by pooling equal amounts of DNA from the 30 individuals with red leaves and the 30 individuals with green leaves.

### Genome sequencing and analysis

BSA-seq was performed on the Illumina NovaSeq platform (BENAGEN, China). The original sequencing data was obtained and subsequently filtered for quality control. Low-quality raw data were filtered using NGSQC toolkit software, and the clean reads from PA21, M84, R-pool, and G-pool were aligned to the perilla reference genome (https://www.ncbi.nlm.nih.gov/genome/?term=PRJNA431002) using GATK software for mutation detection. SAMtools were utilized for single-nucleotide polymorphism (SNP) calling. The SNP index and Δ (SNP index) were calculated to identify candidate regions associated with the riceyness trait. Candidate sites were selected based on the following criteria: the sequencing depth of both parents and progeny pools at the site exceeded the minimum set threshold; the base types between parents were pure and inconsistent; and there was no parent gene present in the progeny. Based on this filtering, the SNP index of the two progeny samples was calculated using one parent as a reference (the SNP index indicates the ratio of reads with different base types to the total reads of the reference genome), and loci with an SNP index less than 0.3 in both progeny were removed. The ΔSNP index (the difference between the SNP indices of the two progeny) was then calculated for all sites. The average ΔSNP index of SNP sites within a sliding window was computed, and the upper and lower boundary thresholds of 95% and 99% confidence intervals obtained through simulation hypothesis testing were combined to screen the candidate regions related to traits. SNP sites in the candidate regions were extracted and annotated. Finally, genes causing start or stop codon mutations, non-synonymous mutations, or variable splicing sites were selected as candidate genes.

### RNA-Seq analysis

Leaf tissue from the parental lines PA21 and M84, as well as from the offspring lines R-pool and G-pool, was collected to isolate total RNA using a Plant RNA Mini Kit (Tiangen, Inc., China). The concentration and quality of total RNA were measured using a NanoDrop 2000 spectrophotometer (Thermo Scientific, Wilmington, DE, United States). Three biological replicates were performed for each sample. Purified RNA was used to construct cDNA libraries with a NEBNext Ultra™ RNA Library Prep Kit for Illumina (NEB, Inc., USA). Library quality was assessed on the Agilent Bioanalyzer 2100 system (Agilent Technologies, Inc., Santa Clara, CA, USA). The four library preparations were subsequently sequenced on an Illumina NovaSeq platform, generating paired-end reads. Data analysis followed the procedures described by Jian et al. (2019). QoRTs (version 1.3.0) software was utilized for quality control and data processing of reads. High-quality reads were mapped to the perilla reference genome sequence using HISAT2; only reads with a perfect match or one mismatch were further analyzed and annotated based on the reference genome. Gene expression was quantified using HTSeq (version 0.11.2) software, and expression levels were estimated by fragments per kilobase of transcript per million fragments mapped (FPKM). Differential expression analysis between the two parental lines was performed using EdgeR (version 3.28.0). The resulting *p*-values were adjusted using the Benjamini and Hochberg approach to control the false discovery rate. Genes with an adjusted *p*-value < 0.05 identified by DESeq were classified as differentially expressed (Love et al. 2014). Significant differentially expressed genes (DEGs) were determined based on a threshold of 1.2-fold expression change. Gene enrichment analysis was conducted using clusterProfiler (version 3.14.3) software.

### Phylogenetic analysis and tree construction

Phylogenetic trees for PfMYB113b and other MYB113 genes were constructed using MEGA7.0 software (Kumar et al. 2016), with sequences from perilla and other species retrieved from the GenBank database (Table S9). Genetic distances were calculated using the p-distance matrix, and evolutionary relationships were inferred using the neighbor-joining method with 1000 bootstrap resampling.

### Quantitative RT-PCR analysis

Quantitative reverse-transcription PCR (qRT-PCR) was conducted following the protocol described by Livak and Schmittgen (Livak & Schmittgen 2001). Total RNA was extracted from the samples using a MAGEN RNA Extraction Kit (MAGEN, USA), and reverse transcription was performed using an Evo M-WLV RT Kit (Accurate, Hunan, China). Gene-specific primers were designed using Primer 5.0, with the primer sequences listed in Table S4. The specificity of the primers was confirmed through agarose gel electrophoresis. PCR amplification was conducted using a LightCycler 480 II REAL-TIME PCR system (Roche, Basel, Switzerland). The qPCR cycling conditions were as follows: pre-denaturation at 95 °C for 30 s, followed by 40 cycles of denaturation at 95 °C for 5 s and annealing/extension at 60 °C for 30 s. The final step included melting curve analysis at 95 °C for 5 s, followed by 60 °C for 1 min. Each gene was amplified in triplicate, with the perilla Actin gene serving as the internal reference gene.

### Yeast one-hybrid (Y1H) assay

The bait vectors pAbAi-PfC4H1pro, pAbAi-PfF3Hpro, and pGADT7-PfMYB113b-JG were constructed. Using the Clontech yeast two-hybrid system, linearized pAbAi-*PfC4H1pro* and pAbAi-*PfF3Hpro* were integrated into the Y1HGold strain and cultured on SD/-Ura plates for 3 to 5 days. Single colonies of Y1HGold containing pAbAi-*PfC4H1pro* and pAbAi-*PfF3Hpro* were selected from the SD/-Ura plates, suspended in a 0.9% NaCl solution to an OD600 of 0.002, and then plated with 100 µl of the bacterial solution in SD/-Ura medium supplemented with various concentrations of AbA (0, 100, 200, 300, 500, 700, 900 ng/ml) for self-activation detection. Based on colony growth, the final screening concentration was determined for subsequent screening experiments. When the AbA concentration was set to 900 ng/ml, the decoy gene was deemed unsuitable for the Y1HGold yeast monohybrid system. Y1HGold cells containing pAbAi-*PfC4H1pro* and pAbAi-*PfF3Hpro* were prepared, and 1.1 × TE/LiAc solution, pre-denatured Carrier DNA, prey plasmid, and DMSO were added successively. The mixture was gently mixed and coated onto SD/-Leu and SD/-Leu/300 AbA plates, then incubated at 30 °C for 3 to 5 days to allow for the growth of positive clones.

## Supplementary Information

Supplementary Material 1. Fig. S1 Simplified scheme for QTL-Seq. F1 offspring were obtained by crossing the high value parent PA21 with the low value parent M84, and then F2 generation isolated population was obtained by F1 generation self-crossbreeding. In this study, the progeny with purple front and back sides were selected to form the highest bulk and the progeny with green front and back sides were selected to form the lowest bulk from the F2 generation for BSA sequencing. Fig. S2 Statistical map of GC content and sequencing depth. a, b, c and d were the statistical maps of GC content and sequencing depth of PA21, M84, A1-pool and A1-pool, respectively. Fig. S3 Delta-snp-indel of 20 chromosomes. SNP-index Manhattan plot of G-pool, R-pool, and Δ (SNP-index) from the BSA analysis. SNP-index graphs of (a, d) highest bulk and (b, e) lowest bulk. (c, f) Δ(SNP-index) graph. The X-axis shows the position of the 20 chromosomes and the Y-axis shows the SNP-index (a, b, d, e) and Δ(SNP-index) (c, f). The blue site indicates that the SNP site corresponds to the ΔSNP-index value between the two progeny. The red line represents the average ΔSNP-index of all SNPS in the sliding window. The green and orange lines represent the upper and lower boundaries of the 95% and 99% confidence intervals, respectively. Fig. S4 Sample phenotype and candidate region of BSR-Seq. (a) Phenotypes of high-value parent P1 (PA21), low-value parent P2 (M84), high-value offspring R-pool, and high-value offspring G-pool. (b) The blue locus indicates that the SNP locus corresponds to the ΔSNP-index value between the two progeny. The red line represents the average ΔSNP-index of all SNPS in the sliding window. The green and orange lines represent the upper and lower boundaries of the 95% and 99% confidence intervals, respectively. Fig. S5 Quantification of BSR-Seq. (a, b) Principal component analysis (PCA) between each group of samples. (c, d) box maps and distribution maps of differentially expressed genes between groups. (e) Scatter plot of correlation between pairwise samples. Fig. S6 Enrichment analysis of BSR-Seq. (a) GO enrichment analysis of differentially expressed transcriptome genes. (b) KEGG enrichment analysis of differentially expressed transcriptome genes. Fig. S7 Functional enrichment analysis of differentially expressed genes between groups. KEGG enrichment analysis of differentially expressed transcriptome genes between M84 vs PA21 (a) and G-pool vs R-pool (b). GO enrichment analysis of differentially expressed transcriptome genes between M84 vs PA21 (b) and G-pool vs R-pool (d). Fig. S8 Identification of PfMYB113b. (a) Successful cloning of CDS sequence of PfMYB113b. (b) Phylogenetic tree of MYB113b protein sequence. The accession number and species origin of each homologous protein sequence are shown in Table S8.

Supplementary Material 2.

## Data Availability

Statistical analysis was conducted using SPSS v20 (IBM Corp., Armonk, NY, USA) for one-way analysis of variance (ANOVA), and GraphPad 8.0.2 software was used for Student's t-test. The following notations were used in the figures: *, *P* < 0.05; **, *P* < 0.01; and ***, *P* < 0.001. Different letters positioned above the bars in the figures indicate significant groupings (*P* < 0.05) determined by ANOVA. The data presented in the figures represent the mean values, while the error bars indicate standard deviations. The materials in this study are availability.
