# Supplementary material for: Molecular mapping of candidate genes in determining red color of perilla leaf
Source: Adv Biotechnol (Singap). 2025 Feb 14;3(1):7. doi: 10.1007/s44307-025-00058-8 (PMC11828775; doi:10.1007/s44307-025-00058-8)
Supplement: Supplementary file 1 — Supplementary Material 1. Fig. S1 Simplified scheme for QTL-Seq. F1 offspring were obtained by crossing the high value parent PA21 with the low value parent M84, and then F2 generation isolated population was obtained by F1 generation self-crossbreeding. In this study, the progeny with purple front and back sides were selected to form the highest bulk and the progeny with green front and back sides were selected to form the lowest bulk from the F2 generation for BSA sequencing. Fig. S2 Statistical map of GC content and sequencing depth. a, b, c and d were the statistical maps of GC content and sequencing depth of PA21, M84, A1-pool and A1-pool, respectively. Fig. S3 Delta-snp-indel of 20 chromosomes. SNP-index Manhattan plot of G-pool, R-pool, and Δ (SNP-index) from the BSA analysis. SNP-index graphs of (a, d) highest bulk and (b, e) lowest bulk. (c, f) Δ(SNP-index) graph. The X-axis shows the position of the 20 chromosomes and the Y-axis shows the SNP-index (a, b, d, e) and Δ(SNP-index) (c, f). The blue site indicates that the SNP site corresponds to the ΔSNP-index value between the two progeny. The red line represents the average ΔSNP-index of all SNPS in the sliding window. The green and orange lines represent the upper and lower boundaries of the 95% and 99% confidence intervals, respectively. Fig. S4 Sample phenotype and candidate region of BSR-Seq. (a) Phenotypes of high-value parent P1 (PA21), low-value parent P2 (M84), high-value offspring R-pool, and high-value offspring G-pool. (b) The blue locus indicates that the SNP locus corresponds to the ΔSNP-index value between the two progeny. The red line represents the average ΔSNP-index of all SNPS in the sliding window. The green and orange lines represent the upper and lower boundaries of the 95% and 99% confidence intervals, respectively. Fig. S5 Quantification of BSR-Seq. (a, b) Principal component analysis (PCA) between each group of samples. (c, d) box maps and distribution maps of differentially expressed [file 44307_2025_58_MOESM1_ESM.pdf]

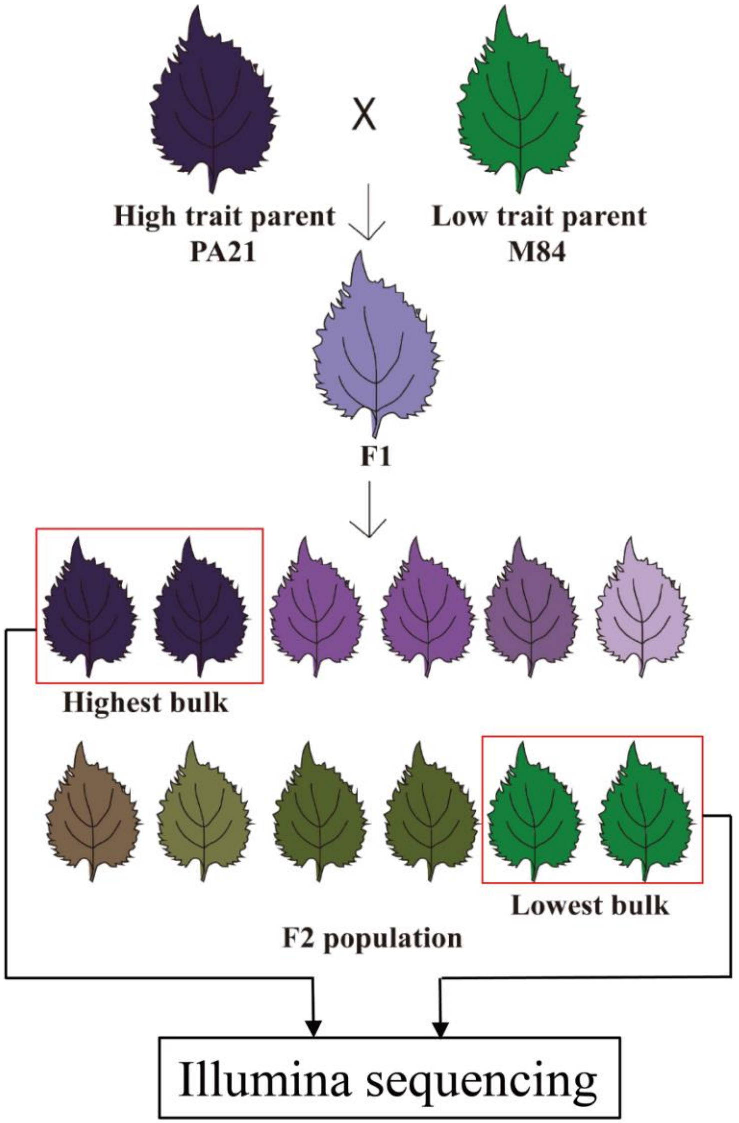

Fig. S1 Simplified scheme for QTL-Seq. F1 offspring were obtained by crossing the high value parent PA21 with the low value parent M84, and then F2 generation isolated population was obtained by F1 generation self-crossbreeding. In this study, the progeny with purple front and back sides were selected to form the highest bulk and the progeny with green front and back sides were selected to form the lowest bulk from the F2 generation for BSA and BSR sequencing.

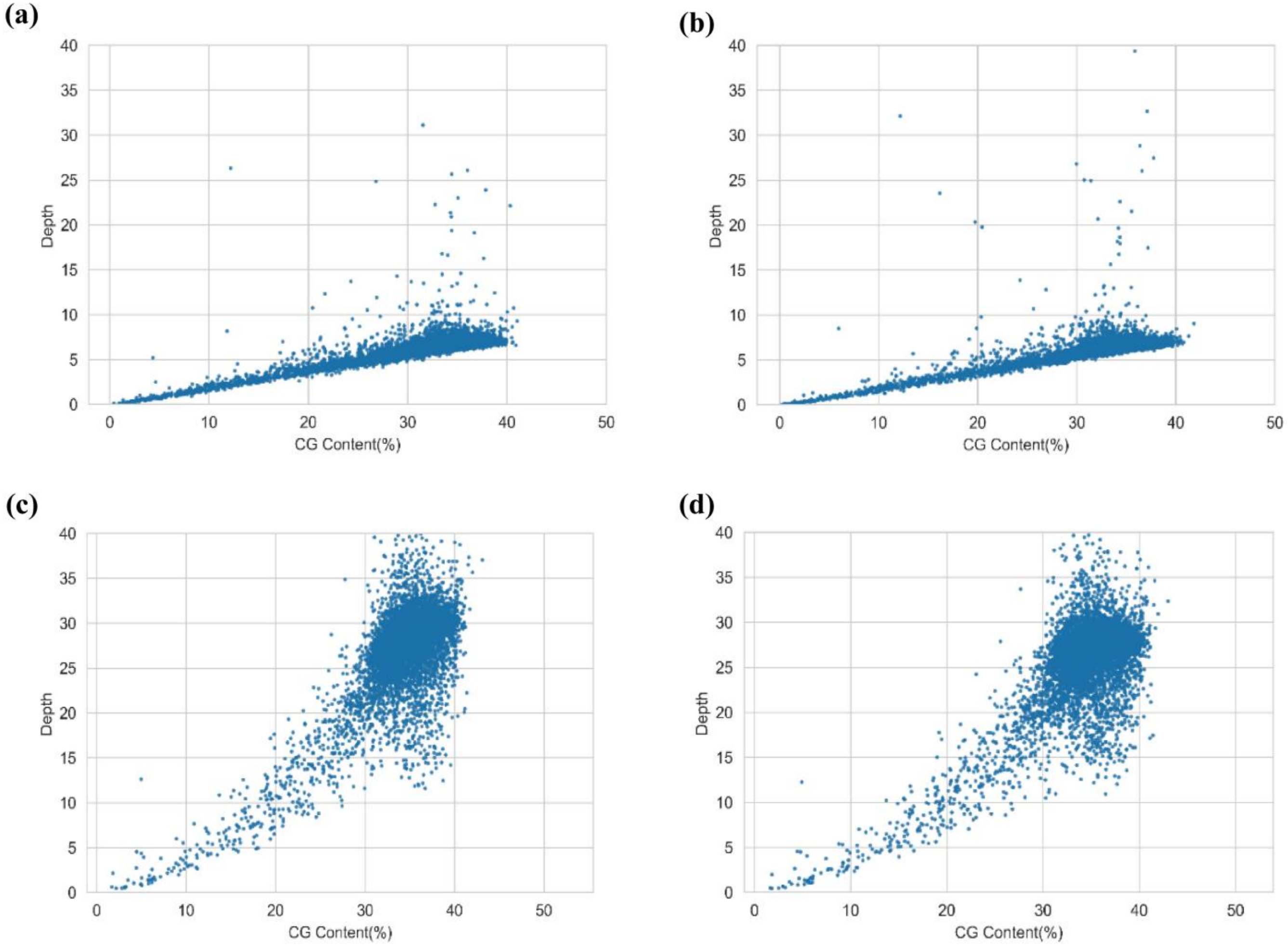

Fig. S2 Statistical map of GC content and sequencing depth. a, b, c and d were the statistical maps of GC content and sequencing depth of PA21, M84, A1-pool and A1-pool, respectively.

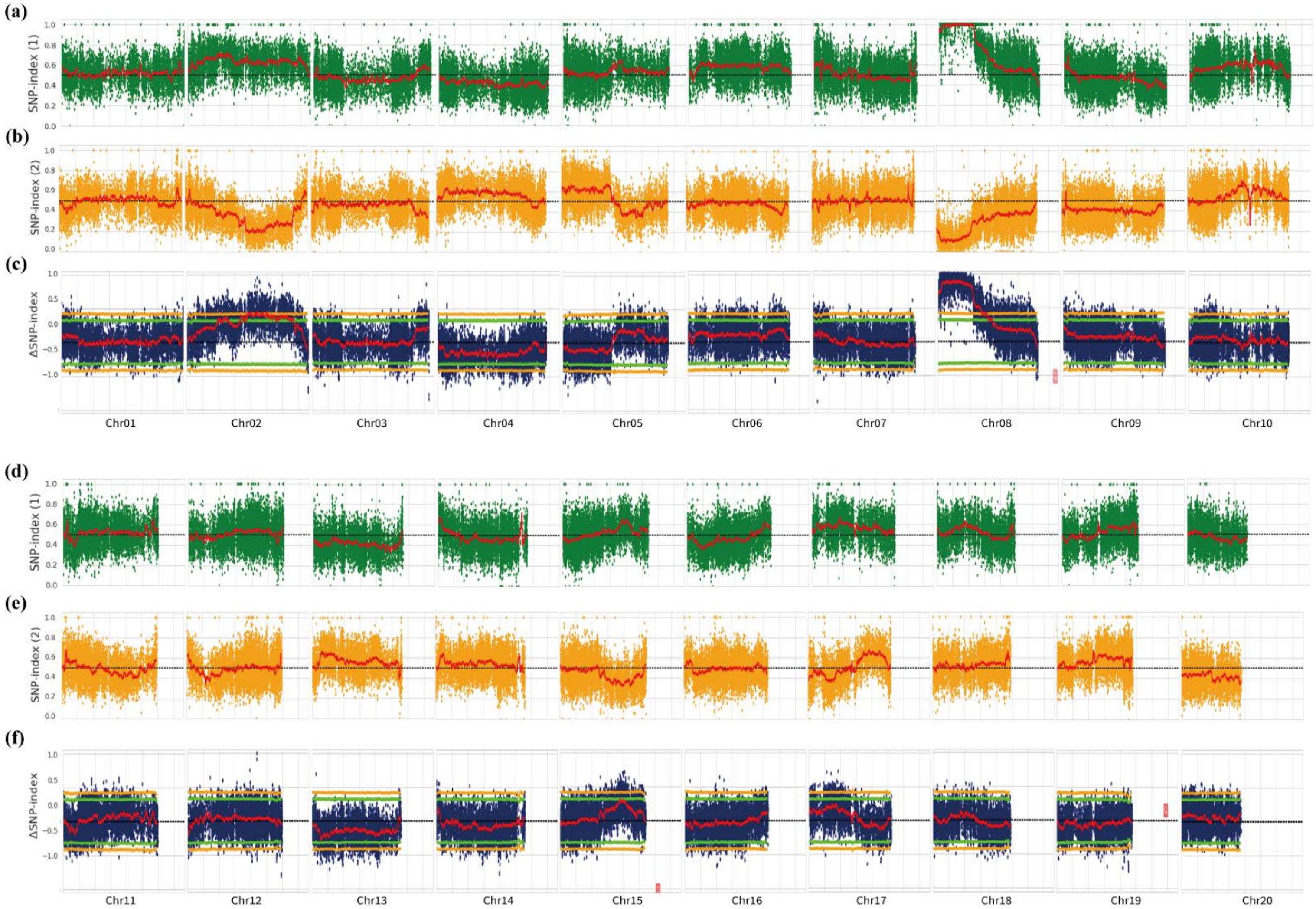

Fig. S3 Delta-snp-indel of 20 chromosomes. SNP-index Manhattan plot of G-pool, R-pool, and Δ (SNP-index) from the BSA analysis. SNP-index graphs of (a, d) highest bulk and (b, c) lowest bulk. The *X*-axis shows the position of the 20 chromosomes and the *Y*-axis shows the SNP-index (a, b, d, e) and Δ(SNP-index) (e, f). The blue site indicates that the SNP site corresponds to the ΔSNP-index value between the two progeny. The red line represents the average ΔSNP-index of all SNPS in the sliding window. The green and orange lines represent the upper and lower boundaries of the 95% and 99% confidence intervals, respectively.

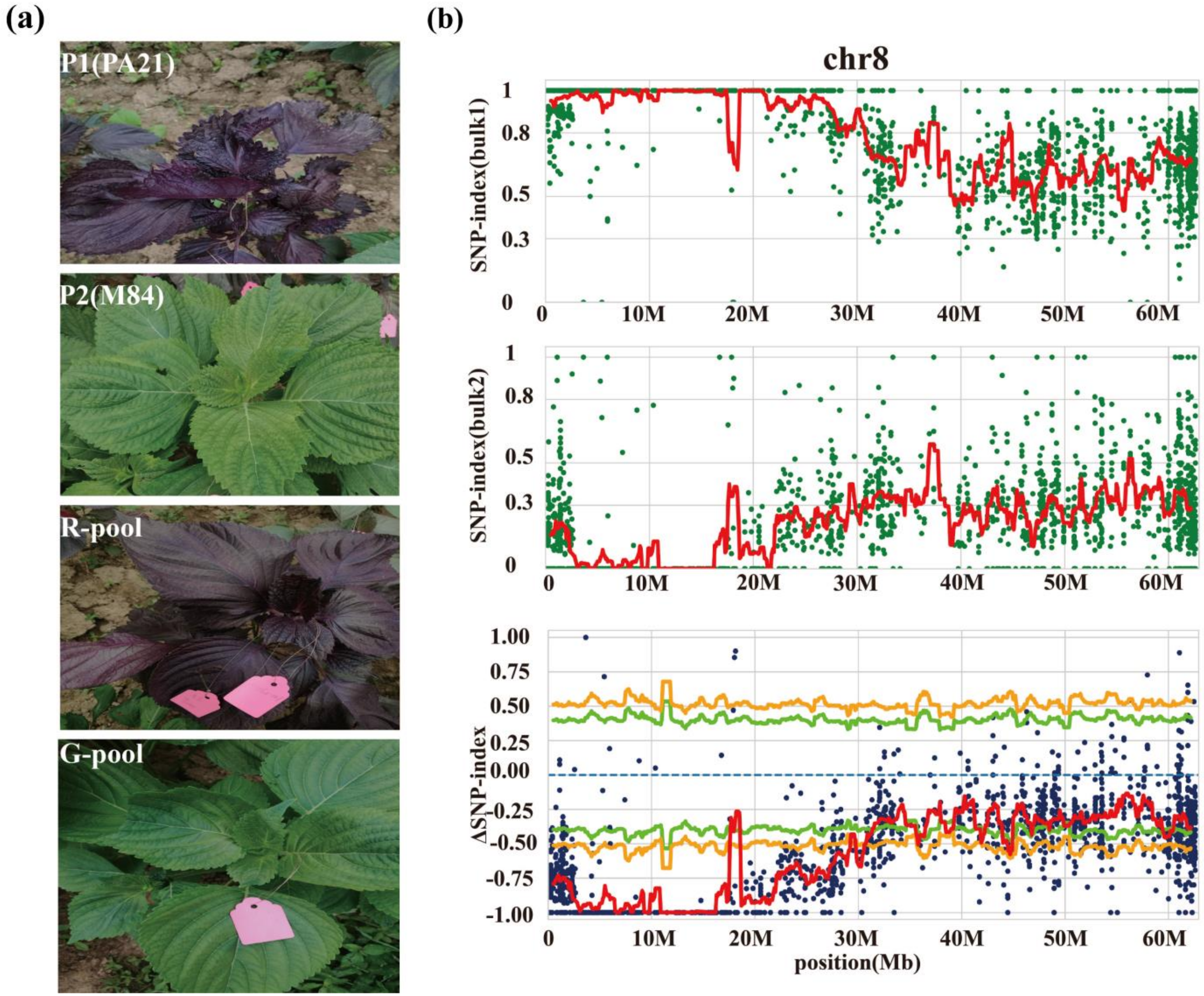

Fig. S4 Sample phenotype and candidate region of BSR-Seq. (a) Phenotypes of high-value parent P1 (PA21), low-value parent P2 (M84), high-value offspring R-pool, and high-value offspring G-pool. (b) The blue locus indicates that the SNP locus corresponds to the ΔSNP-index value between the two progeny. The red line represents the average ΔSNP-index of all SNPS in the sliding window. The green and orange lines represent the upper and lower boundaries of the 95% and 99% confidence intervals, respectively.

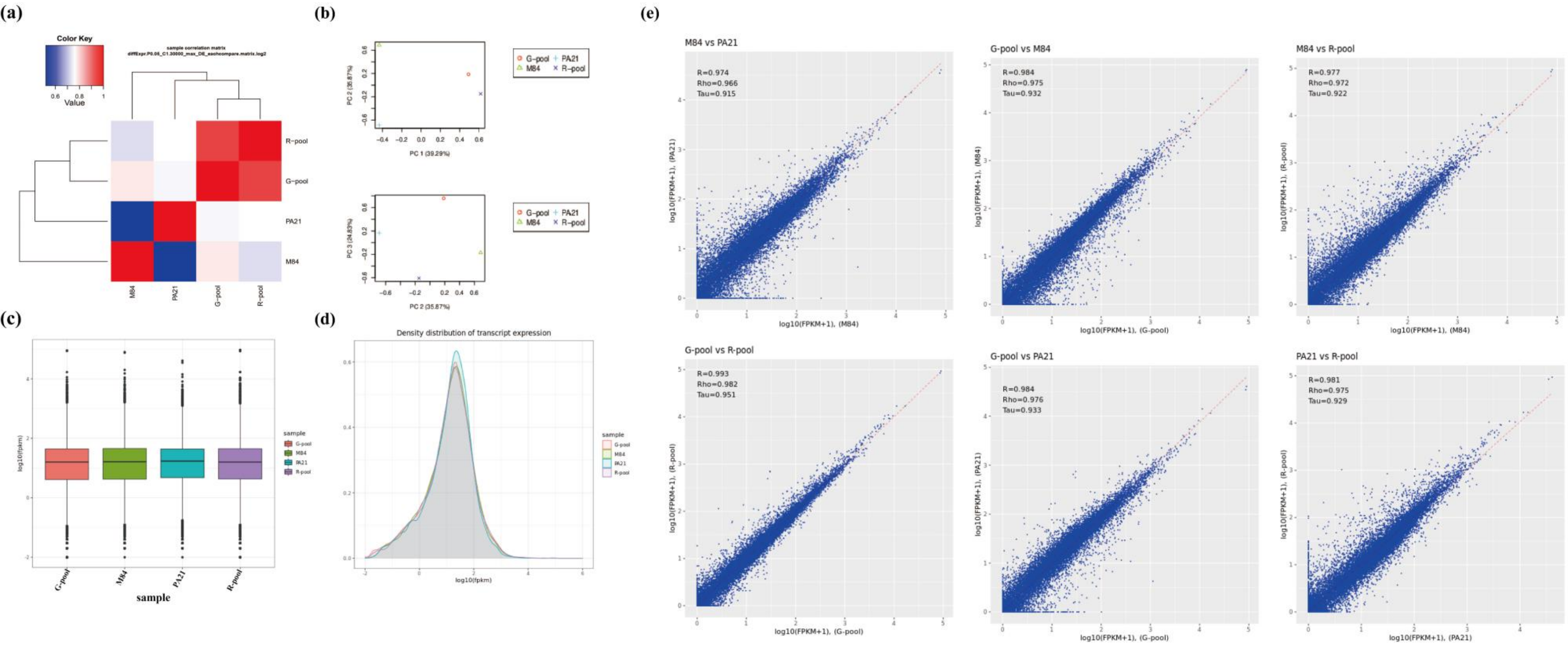

Fig. S5 quantification of BSR-Seq. (a, b) Principal component analysis (PCA) between each group of samples. (c, d) box maps and distribution maps of differentially expressed genes between groups. (e) Scatter plot of correlation between pairwise samples.

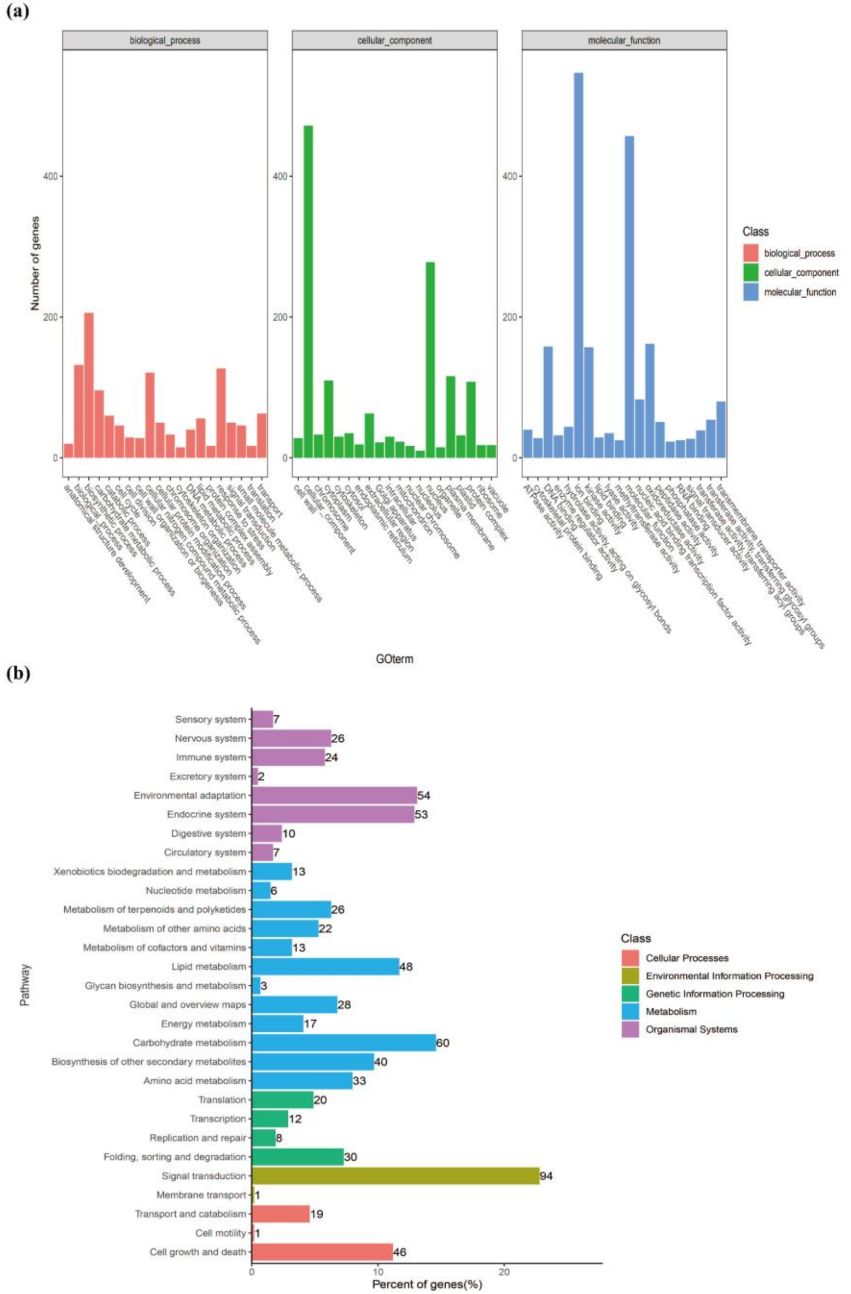

Fig. S6 Enrichment analysis of BSR-Seq. (a) GO enrichment analysis of differentially expressed transcriptome genes. (b) KEGG enrichment analysis of differentially expressed transcriptome genes.

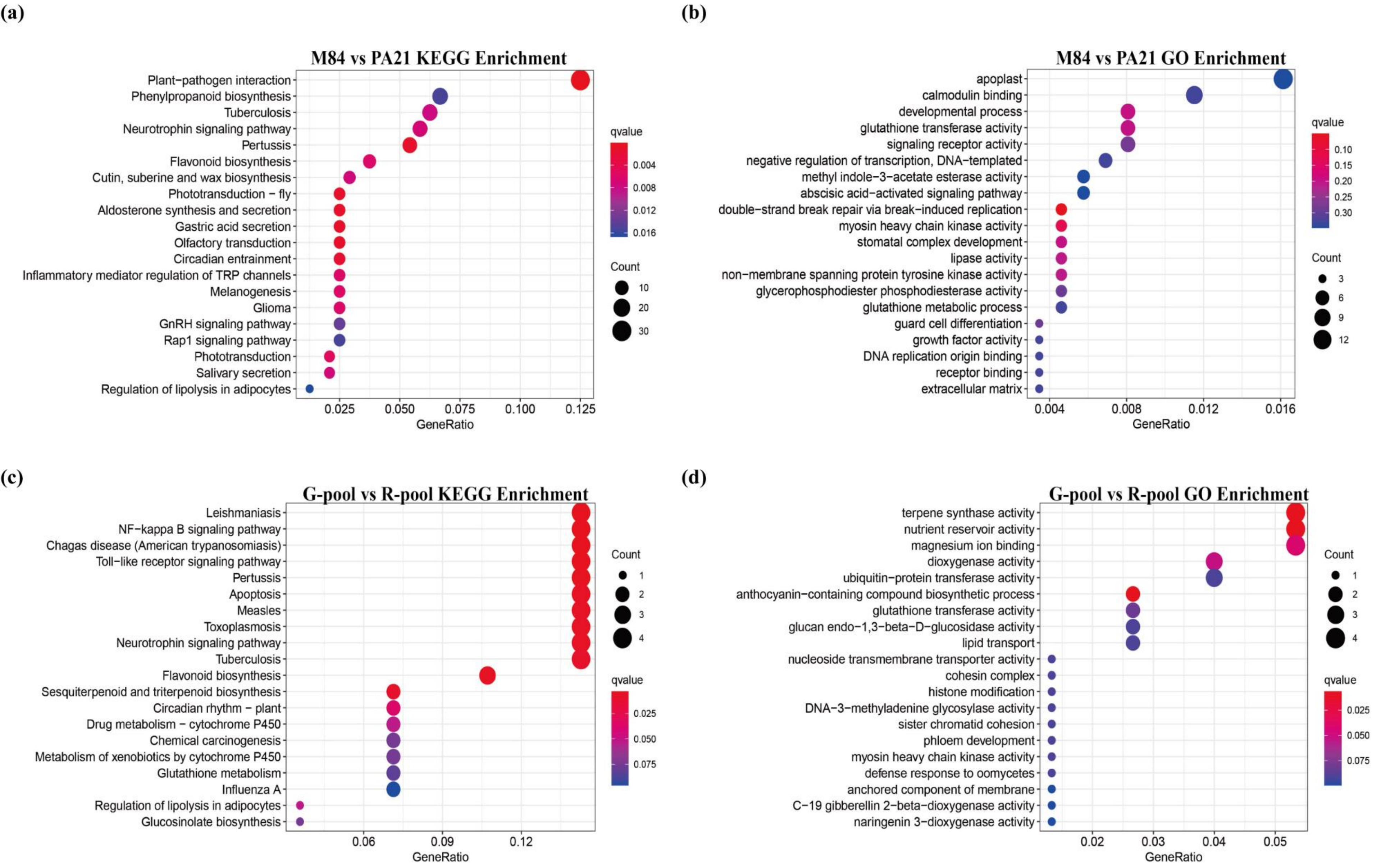

Fig. S7 Functional enrichment analysis of differentially expressed genes between groups. KEGG enrichment analysis of differentially expressed transcriptome genes between M84 vs PA21 (a) and G-pool vs R-pool (c). GO enrichment analysis of differentially expressed transcriptome genes between M84 vs PA21 (b) and G-pool vs R-pool (d).

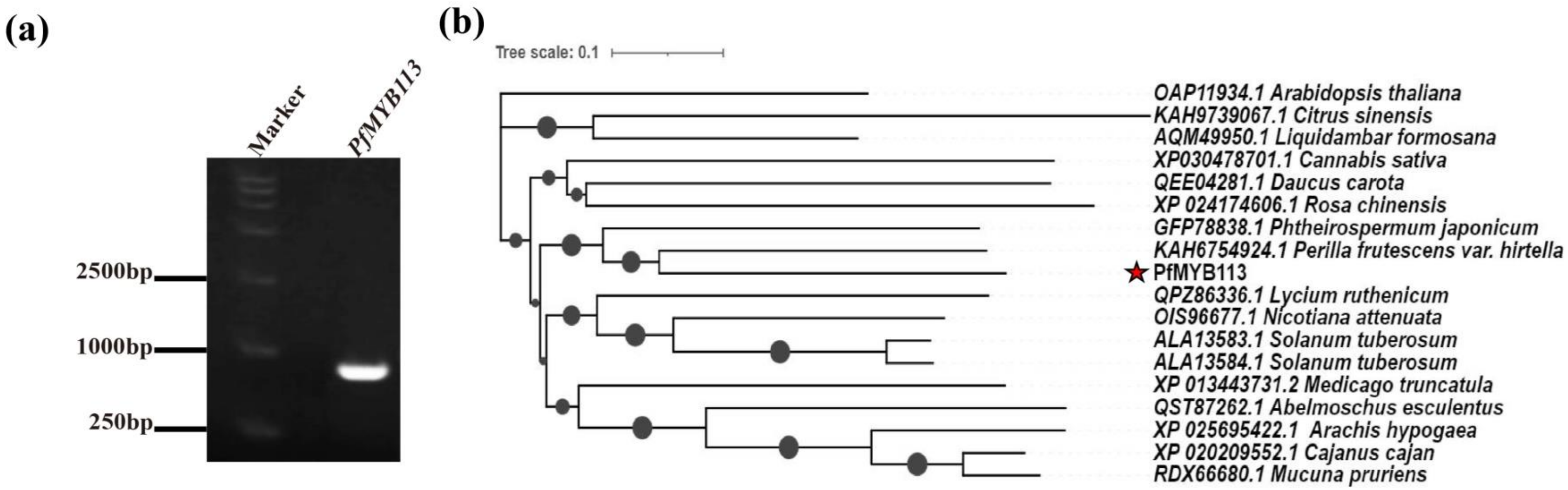

Fig. S8 Identification of *PfMYB113b*. (a) Successful cloning of CDS sequence of *PfMYB113b*. (b) Phylogenetic tree of *MYB113b* protein sequence.
