# Supplementary material for: Molecular mapping of candidate genes in determining red color of perilla leaf
Source: Adv Biotechnol (Singap). 2025 Feb 14;3(1):7. doi: 10.1007/s44307-025-00058-8 (PMC11828775; doi:10.1007/s44307-025-00058-8)
Supplement: Supplementary file 2 — Supplementary Material 2. [file 44307_2025_58_MOESM2_ESM.pdf]

Table S1 BSA-Seq Data quality statistics

Population	Sample	Raw Reads	Clean Reads	Raw Base （G）	Clean Base （G）	Q20 （%）	Q30 （%）	GC Content （%）
M84 x PA21	M84	34032651	33890652	10.21	10.17	97.5	92.78	35.91
	PA21	34083295	33940766	10.22	10.18	96.59	90.94	35.94
	R-pool	156271321	155697263	46.88	46.71	96.48	90.64	36.05
	G-pool	146820772	146163528	44.05	43.85	96.56	90.84	35.69

R-pool: high value F2 generation population; G-pool: low value F2 generation population; Sample: Name of the sample; Raw reads: The number of sequencing Raw reads; Clean reads: Number of Clean reads obtained after filtration; Raw bases: The total bases of the Raw data are calculated by multiplying the number of Raw reads by the sequencing length, in G. Q20, Q30: The percentage of bases with Phred values greater than 20 and 30 in the total base; GC: The percentage of G/C bases in the total number of bases.

Table S2 Statistical table of BSA data comparison results

Population	Sample	Rawbases	Total Reads	Mapped Rate	Depth(X)	Coverage Rate
M84 x PA21	M84	10.21	58910580	99.52%	7.08507	95.06%
	PA21	10.22	60719500	99.23%	7.26472	93.92%
	R-pool	46.88	261496188	99.05%	31.0035	97.73%
	G-pool	44.05	247347643	98.77%	29.2954	97.7%

Sample: sample name; Total reads: The total number of reads after weight removal was compared. Mapped rate: reads to the reference genome; Depth: sequencing depth; Coverage rate: sequence coverage.

Table S3 SNP and Indel statistics of parental and mixed-pool resequencing

Sample	SNP	Indel
M84	2,599,994	581,580
PA21	4,246,282	833,075
A1	6,293,712	1,281,990
A2	6,230,345	1,268,291

Table S4 SNP variation types in BSA results

Types	SNP Num.	Gene Num.
missense_variant	122	101
synonymous_variant	62	59
disruptive_inframe_deletion	1	1
splice_region_variant& intron_variant	11	11
intron_variant	500	244
frameshift_variant&stop_gained	1	1
conservative_inframe_deletion	3	3
frameshift_variant	3	2
disruptive_inframe_insertion	1	1
stop_gained	1	1
splice_donor_variant&intron_variant	2	2
splice_region_variant&synonymous_variant	1	1

Table S5 Data quality control statistics for BSR-seq

Sample	Raw reads	Raw bases	Clean reads	Clean bases	Q20_rate	Q30_rate
PA21	44570008	6685501200	44334586	6626276484	98.016%	94.117%
M84	50317200	7547580000	50080098	7409547972	98.032%	94.157%
R-pool	79290228	11893534200	78859504	11782624208	98.053%	94.283%
G-pool	92024156	13803623400	91513816	13662208848	97.712%	93.388%

Sample: Sample number; Raw reads: Total number of sequenced reads; Raw bases: total number of bases of the sequence; Clean reads: Total number of filtered reads; Clean bases: Total bases of the sequence after filtration; Q20_rate: The ratio of filtered base mass greater than Q20; Q30_rate: The filtered base mass is greater than Q30 ratio.

Table S6 Statistical table of BSR-seq data comparison results

Sample	total Reads	mapped Reads	mapped rate	depth	coverage
PA21	44,334,586	44,274,359	99.86%	5.03774	10.089%
M84	50,080,098	50,029,519	99.90%	5.43598	9.164%
G-pool	91,513,816	91,412,178	99.89%	9.51105	10.786%
R-pool	78,859,504	78,762,951	99.88%	8.08522	10.010%

Sample: total Reads: The number of reads after weight removal. mapped Reads: total number of reads compared; mapped rate: reads to the reference genome; depth: sequencing depth; coverage: Sequence coverage.

Table S7 Information of Candidate Genes in BSA-Seq

Genes ID	Annotation information
chr08_09912247_09913840	Cytochrome p450（F3'H/CYP736A12）
chr08_10077188_10078763	Cytochrome p450（F3'H/CYP736A12）
chr08_15793356_15797718	Phenylalanine ammonia-lyase
chr08_09322101_09322880	ethylene-responsive transcription factor
chr08_09517363_09521707	Agamous-like MADS-box protein
chr08_09522310_09525473	Transcription factor
chr08_09760251_09761921	TGACG-sequence-specific DNA-binding protein
chr08_11487857_11491178	Myb-like DNA-binding domain（PfMYB113）
chr08_11789666_11790252	homeobox-leucine zipper protein
chr08_11849337_11851039	WRKY Transcription Factor
chr08_12452534_12460724	Histone-lysine N-methyltransferase
chr08_12581327_12583834	helix loop helix domain
chr08_12705469_12707800	protein FAR1-RELATED SEQUENCE
chr08_13175072_13178276	Heat shock factor
chr08_14021369_14036630	SNF2 family N-terminal domain
chr08_14628926_14632725	Histone-like transcription factor
chr08_14695770_14701054	RING/Ubox like zinc-binding domain
chr08_14704612_14707751	Ethylene-responsive transcription factor RAP2-7-like isoform X1
chr08_15495017_15497451	Protein FAR1-RELATED SEQUENCE 5-like
chr08_15625060_15626412	Transcriptional activator that specifically binds 5'-GATA-3' or 5'-GAT-3' motifs within gene promoters

Table S8 Sequence information of homologous proteins in evolutionary tree

Accession number	Species origin
XP_013443731.2	Medicago truncatula
ALA13584.1	Solanum tuberosum
ALA13583.1	Solanum tuberosum
XP_024174606.1	Rosa chinensis
GFP78838.1	Phtheirospermum japonicum
OIS96677.1	Nicotiana attenuata
RDX66680.1	Mucuna pruriens
AQM49950.1	Liquidambar formosana
KAH9739067.1	Citrus sinensis
XP_030478701.1	Cannabis sativa
XP_020209552.1	Cajanus cajan
XP_025695422.1	Arachis hypogaea
OAP11934.1	Arabidopsis thaliana
QST87262.1	Abelmoschus esculentus
QPZ86336.1	Lycium ruthenicum
QEE04281.1	Daucus carota
PfMYB113	Perilla frutescens var. frutescens
KAH6754924.1	Perilla frutescens var. hirtella

Table S9 Information of PfC4H1, PfF3H and PfMYB113b genes

Gene	GeneID	G-pool	M84	PA21	R-pool
PfC4H1	chr12_09750325_09752237	145.29	76.76	522.6	489.7
PfF3H	chr19_41462565_41466468	1.71	0.77	82.18	113.2
PfMYB113b	chr08_11487857_11491178	22.47	26.27	185.53	160.42
